# Impaired Auditory-Vestibular Functions and Behavioral Abnormalities of *Slitrk6*-Deficient Mice

**DOI:** 10.1371/journal.pone.0016497

**Published:** 2011-01-26

**Authors:** Yoshifumi Matsumoto, Kei-ichi Katayama, Takehito Okamoto, Kazuyuki Yamada, Noriko Takashima, Soichi Nagao, Jun Aruga

**Affiliations:** 1 Laboratory for Behavioral and Developmental Disorders, RIKEN Brain Science Institute (BSI), Wako-shi, Japan; 2 Laboratory for Motor Learning Control, RIKEN Brain Science Institute (BSI), Wako-shi, Japan; 3 Support Unit for Animal Experiments, RIKEN Brain Science Institute (BSI), Wako-shi, Japan; The Research Center of Neurobiology-Neurophysiology of Marseille, France

## Abstract

A recent study revealed that Slitrk6, a transmembrane protein containing a leucine-rich repeat domain, has a critical role in the development of the inner ear neural circuit. However, it is still unknown how the absence of *Slitrk6* affects auditory and vestibular functions. In addition, the role of *Slitrk6* in regions of the central nervous system, including the dorsal thalamus, has not been addressed. To understand the physiological role of Slitrk6, *Slitrk6*-knockout (KO) mice were subjected to systematic behavioral analyses including auditory and vestibular function tests. Compared to wild-type mice, the auditory brainstem response (ABR) of *Slitrk6*-KO mice indicated a mid-frequency range (8–16 kHz) hearing loss and reduction of the first ABR wave. The auditory startle response was also reduced. A vestibulo-ocular reflex (VOR) test showed decreased vertical (head movement–induced) VOR gains and normal horizontal VOR. In an open field test, locomotor activity was reduced; the tendency to be in the center region was increased, but only in the first 5 min of the test, indicating altered adaptive responses to a novel environment. Altered adaptive responses were also found in a hole-board test in which head-dip behavior was increased and advanced. Aside from these abnormalities, no clear abnormalities were noted in the mood, anxiety, learning, spatial memory, or fear memory–related behavioral tests. These results indicate that the *Slitrk6*-KO mouse can serve as a model of hereditary sensorineural deafness. Furthermore, the altered responses of *Slitrk6*-KO mice to the novel environment suggest a role of *Slitrk6* in some cognitive functions.

## Introduction

Slitrk6 belongs to the Slitrk family of transmembrane proteins with neurite outgrowth modulating activities [1], [2]. Structurally, Slitrk members share two leucine-rich repeat domains located amino-terminal to the transmembrane domain. In the carboxy-terminus, there are conserved tyrosine residues flanked by amino acid sequences similar to those in the carboxy-terminal domain of the Ntrk neurotrophin receptor [3]. Among the six *Slitrk* family genes (*Slitrk1–6*) in mice, *Slitrk6* shows a unique expression pattern, with strong expression in the inner ear and modest expression in the dorsal thalamus at both embryonic and postnatal stages [1], [4]–[6].

A recent study revealed that Slitrk6 promotes innervation and survival of inner ear sensory neurons in part by modulating neurotrophin–Ntrk signaling [5]. *Slitrk6*-knockout (KO) mice showed reduced cochlear innervation. In the vestibule, the innervation to the posterior crista was often lost, reduced, or sometimes misguided. These defects were accompanied by the loss of neurons in the spiral and vestibular ganglia. This study addressed only the embryonic to early postnatal morphological phenotype of *Slitrk6-*KO mice, and analyses of any functional deficits caused by the developmental defects of the inner ear remain to be conducted.

Previous studies reported Slitrk family members as candidate genes controlling several neuropsychiatric disorders [7]–[13]. Considering the expression of *Slitrk6* in the mature central nervous system [1], [2] (http://www.brain-map.org/), it is possible that *Slitrk6* has a role in the expression of higher brain functions.

In the present study, we evaluated the neurological characteristics of the *Slitrk6*-KO mouse by performing systematic behavioral analyses and physiological tests related to inner ear functions. The results revealed deficits in the auditory, vestibular, and cognitive functions of *Slitrk6*-KO mice. We discussed the biological significance of *Slitrk6* and the similarities of deficits in the KO mice to the phenotypes of some neurological diseases.

## Results

### General features of the *Slitrk6*-KO mouse

The *Slitrk6* null allele (*Slitrk6^−^*) was generated by replacing its entire protein-coding region by a loxP sequence [5]. Both male and female *Slitrk6*-KO (*Slitrk6^−/−^*) mice grew without showing any external abnormalities and were fertile [5]. A slight, but significant reduction in body weight was observed in the adult male mice (wild type [WT]: *n* = 10, 26.5±0.57 g [mean ± SE]; KO: *n* = 10, 24.6±0.19 g at 12 weeks old). However, the reason for this weight reduction is not clear. We used adult male *Slitrk6*-KO mice and WT (*Slitrk6^+/+^*) littermates for the behavioral and inner ear function tests. The behavioral tests are listed in Table 1.

**Table 1:** Summary of Slitrk6 KO behavioral analyses.

Test	Parameter	Comparison to WT mice
Home cage activity	whole day	n.s.
	light phase	increment in KO mice (08:00–09:00, *P<*0.05)
	dark phase	decrement in KO mice (23:00–01:00, *P* = 0.68)
Open field	total distance	decrement in KO mice (first 5 min, *P<*0.01)
	moving speed	decrement in KO mice (*P<*0.01)
	time in center area (%)	increment in KO mice (first 5 min, *P<*0.01)
Hole-board test	active time	n.s.
	total distance (cm)	n.s.
	head-dip latency (s)	decrement in KO mice (*P<*0.05)
	number of head dips	n.s.
	head dipping duration (s)	increment in KO mice (*P<*0.05)
	rearing duration (s)	n.s.
	number of rearing episodes	n.s.
Elevated plus maze	total distance (cm)	n.s.
	entry number	n.s.
	time in open arm (%)	n.s.
	number of entry to open arm (%)	n.s.
Light–dark box	total distance (cm)	n.s.
	number of transitions	n.s.
	latency to transition to dark box	n.s.
	distance travelled in light box (%)	n.s.
	time spent in light box (%)	n.s.
Morris water maze	total distance (cm)	n.s.
	movement time (s)	n.s.
	latency to platform (s)	n.s.
Fear conditioning	context test	n.s.
	cued test	n.s.
Hot plate test	lick	n.s.
Tail flick test	flinch	n.s.
	jump	n.s.
Rota- rod test	rotation	n.s.
Startle response	startle response	decrement in KO mice (95–120 dB, *P<*0.01)
	initial/final	n.s.
	prepulse inhibition	n.s.
Social interaction	number of contacts	n.s.
Tail suspension	immobility time (%)	decrement in KO mice (*P<*0.01)
Forced swimming	immobility time (%)	n.s.

### Auditory function defects in *Slitrk6*-KO mice

**Figure 1:**
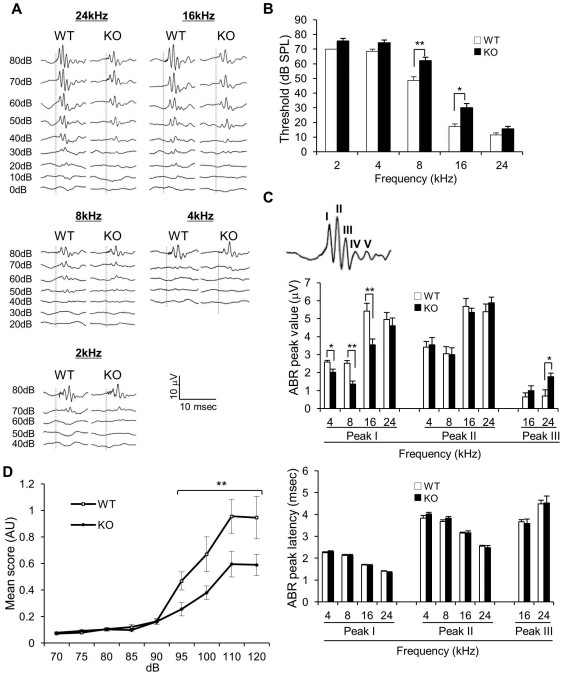
Auditory function abnormalities in *Slitrk6*-KO mice. (A) Representative waves of auditory brainstem response (ABR) from WT (left, *n* = 7) and *Slitrk6*-KO (right, *n* = 9) mice. ABR were recorded upon 0- to 80-dB sound pressure level (SPL) stimuli of 2, 4, 8, 16, and 24 kHz. The dash lines indicate the time of click presentation. The scale bars are shown at bottom right. (B) ABR thresholds in WT and *Slitrk6*-KO mice. *Slitrk6*-KO mice showed significantly higher thresholds to the 8- and 16-kHz stimuli than those of WT mice. (C) *Top:* Each peak number in a representative ABR wave. *Middle:* Comparison of the values of peaks I, II, and III between WT and *Slitrk6*-KO mice. Peak I of *Slitrk6*-KO mice was significantly reduced in the range of 8 to 16 kHz, and peak III was also significantly reduced at 24 kHz. *Bottom:* The latency to peaks I, II, and III. The latency did not show clear differences between WT and *Slitrk6*-KO mice. (D) Auditory startle response of WT (*n* = 10) and *Slitrk6*-KO mice (*n* = 10). *Slitrk6*-KO mice showed significantly lower startle responses to 95- to 120-dB sounds. *P<0.05, **P<0.01. All bars are mean ± standard error of the mean.

Auditory brain-stem response (ABR) is an electrical signal reflecting the neuronal activities related to auditory information processing. The ABR method has been shown to be effective for assessing auditory function in mice [14]. We recorded ABRs in anesthetized mice with ear and scalp electrodes. The responses were recorded upon delivering sound stimuli with certain ranges of frequencies (2–24 kHz) and strength (10–80 dB). Figure 1A presents the ABR waves from WT and *Slitrk6*-KO mice. To determine the hearing function of *Slitrk6*-KO mice, we first measured the thresholds for the appearance of a recognizable wave representing ABR (Fig. 1B). The results indicated significant increments (ca. 20 dB) of the sound intensity threshold for 8-kHz (WT  = 48.5±2.6 [mean ± SE], KO  = 62.2±2.22; *P*<0.01, Mann-Whitney's *U-*test) and 16-kHz (WT  = 17.1±1.84, KO  = 30±2.88; *P*<0.05) stimuli, but not for 2-, 4-, and 24-kHz stimuli at 4 weeks of age. When the amplitude and latency values of peaks I, II, and III with an 80-dB stimulus were compared between WT and *Slitrk6*-KO mice (Fig. 1C top), there was a significant decrement in the peak I absolute values of 16-kHz (WT  = 5.42±0.43 [mean ± SE], KO  = 3.55±0.31; *P* = 0.0031), 8-kHz (WT  = 2.51±0.15, KO  = 1.36±0.16; *P* = 0.00020), and 4-kHz (WT  = 2.58±0.09, KO  = 2.02±0.16; *P* = 0.012) stimuli-induced responses in *Slitrk6*-KO mice (Fig. 1C middle). There also was a significant increment in the peak III absolute values for the 24-kHz stimulus (Fig. 1C middle; WT  = 0.69±0.34, KO  = 1.76±0.20; *P* = 0.015). However, the peak latency was not significantly different between the two genotypes (Fig. 1C bottom).

Auditory startle response (ASR) was included in the auditory function analysis. We measured the ASR following white noise stimulation with various sound intensities (Fig. 1D; 70–120 dB on 65-dB background noise). Compared with WT mice, *Slitrk6*-KO mice showed significant decrements of ASR toward the stronger sound stimuli (95–120 dB; *F*_1,18_ = 9.7, *P* = 0.0059, two-way ANOVA, main effect of genotype) but not toward the weaker ones (70–90 dB). While there was a clear difference in ASR between the genotypes, there were no significant differences in the prepulse (70, 75, and 80 dB) inhibition of ASR induced by a 120-dB stimulus (Table 1). Thus, both ABR and ASR results indicated that there was an auditory function deficit in the *Slitrk6*-KO mice.

### Vertical vestibular function defects in *Slitrk6*-KO mice

**Figure 2:**
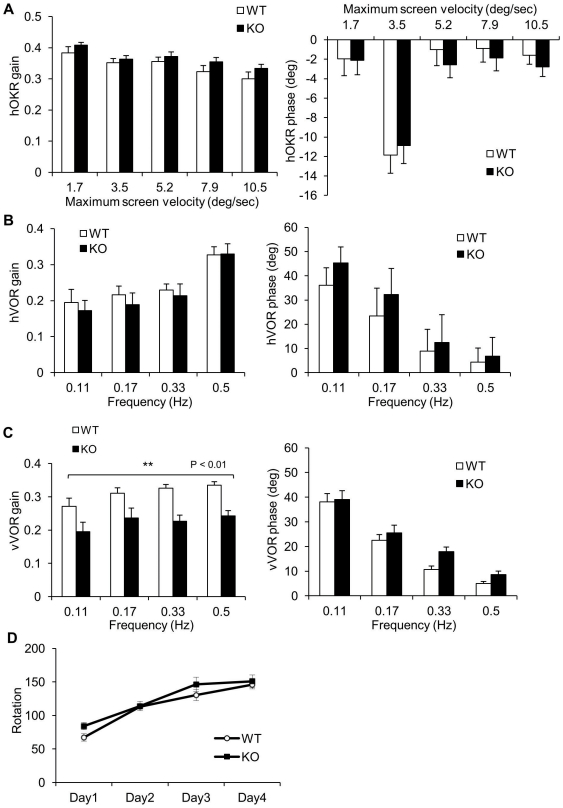
Vestibular function anomaly in *Slitrk6*-KO mice. (A) Dynamic characteristics of horizontal optokinetic response (hOKR) of WT (*n* = 9) and *Slitrk6*-KO (*n* = 9) mice. (B) Horizontal vestibulo-ocular reflex (hVOR). There were no differences in gains (left) or phases (right) between WT (*n* = 9) and KO (*n* = 9) in hVOR or hOKR. (C) Vertical vestibulo-ocular reflex (vVOR) of WT (*n* = 8) and *Slitrk6*-KO mice (*n* = 6). Gains of vVOR (left) of the *Slitrk6*-KO mice were significantly smaller than those of WT. (D) Rota- rod test. Rotation indicates the speed of rotation (rpm) at which the mice fell off or revolved around the rod. The values were comparable between WT (*n* = 12) and *Slitrk6*-KO (*n* = 8) mice, suggesting that there were no strong deficits of balancing function in *Slitrk6*-KO mice. Values are mean ± standard error of the mean.

A previous study revealed the frequent absence of vestibular nerve projection to the posterior crista in *Slitrk6*-KO mice [5]. Therefore, functions of the semicircular canal were investigated. We measured eye movements induced by rotation of the head on the plane of the horizontal semicircular canal, that is, the horizontal vestibulo-ocular reflex (hVOR), or on the plane perpendicular to the plane of the horizontal semicircular canal, the vertical vestibulo-ocular reflex (vVOR). The horizontal optokinetic response (hOKR), which shares the same neural circuit within the cerebellum as that of the hVOR, was measured as a reference. There were no clear differences in the hVOR or hOKR gains and phases between WT and *Slitrk6-*KO mice (Fig. 2A, B). For the vVOR, however, *Slitrk6*-KO mice showed significantly decreased gains at all four frequencies examined (Fig. 2C; *F*_1,12_ = 11.7, *P* = 0.0051, two-way ANOVA, main effect of genotype). There were no significant differences in the vVOR phases. These results indicated that there was a functional deficit in the semicircular canal of *Slitrk6*-KO mice.

We also performed behavioral tests related to vestibular functions, such as swimming, contact righting, and suspension [15]. In the suspension test, we did not see the trunk curling posture that is typically found in mice with impaired vestibular function [15] (data not shown). Swimming and contact righting did not show clear differences between the two genotypes (data not shown). In addition, *Slitrk6*-KO mice displayed a comparable performance to that of WT mice in the rota-rod test (Fig. 2D), which partly reflect vestibular function. These results suggested that the vestibular functional deficit in *Slitrk6-*KO mice is mild and selective for the posterior semicircular canal system.

### Adaptive responses to environmental change are altered in *Slitrk6*-KO mice

**Figure 3:**
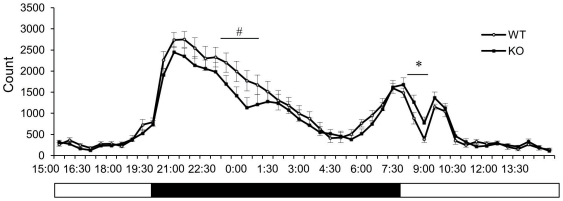
Spontaneous activity in home cage. Each vertical bar represents standard error of the mean. ^#^ *P* = 0.068; *P<0.05. WT, *n* = 10; KO, *n* = 10. Below the graph, open and black bars indicate light phase (08:00–20:00) and dark phase (20:00–08:00), respectively.

In addition to inner ear–related abnormalities in *Slitrk6-*KO mice, novel phenotypes became clear based on their performance in a battery of behavioral tests (Table 1). First, we noticed altered activities of *Slitrk6-*KO mice in the home cage (Fig. 3). The activity level during the early dark phase tended to be lower (20:00–01:00, *P* = 0.11; 23:00–01:00, *P* = 0.068) and that during the beginning of the light phase was significantly higher (08:00–09:00, *P* = 0.045) than activity levels of the WT mice.

**Figure 4:**
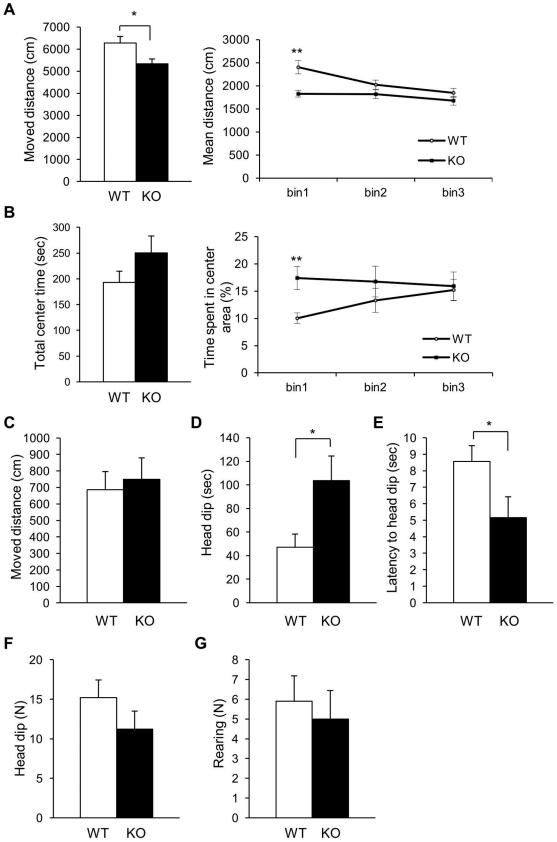
Altered adaptive responses to a novel environment in *Slitrk6*-KO mice. (A, B) Open field test. (A) The total distance moved was less in *Slitrk6*-KO than in WT mice (left). The distance moved in the first 5 min of the test was significantly lower in *Slitrk6*-KO mice (right). (B) In *Slitrk6*-KO mice, the total time spent in the center area was greater than that of the WT (left) and was significantly increased in the first 5 min of the test (right). (C–G) Hole-board test. There were no significant differences in distance moved (C), number of rearing episodes (D), and number of head dips (E) between *Slitrk6*-KO and WT mice. Duration per head dip was significantly decreased in *Slitrk6*-KO mice (F). Latency time to head dip was significantly decreased in KO mice (G). Values are mean ± standard error of the mean. *P<0.05, **P<0.01 for Mann-Whitney's *U*-test (B, center area data) or Student's *t*-test (others). WT, *n* = 10; KO, *n* = 10.

In an open field (OF) test with a 15-min observation period, the *Slitrk6*-KO mice showed less locomotor activity (Fig. 4A), although the difference was limited to the first 5 min (Fig. 4A; *P* = 0.0023). Furthermore, the percentage of time the *Slitrk6*-KO mice remained in the center area was significantly longer than the WT only in the first 5 min (Fig. 4B; *P* = 0.0082, Mann-Whitney's *U*-test). The abnormalities only in the early phase were thought to reflect some altered adaptive responses to the novel environment.

We also noted altered responses to a novel environment in the hole-board (HB) test, which was carried out for 5 min in a box that was the same size as the OF box. However, the HB box differed from the OF box in terms of color, brightness, and the presence of four holes on its floor. The total duration of head dipping into the holes by *Slitrk6*-KO mice was more than twice that of WT (Fig. 4D; *P* = 0.046). In addition, the latency to the first head dip was significantly shorter than that in the WT (Fig. 4E; *P* = 0.028).

**Figure 5:**
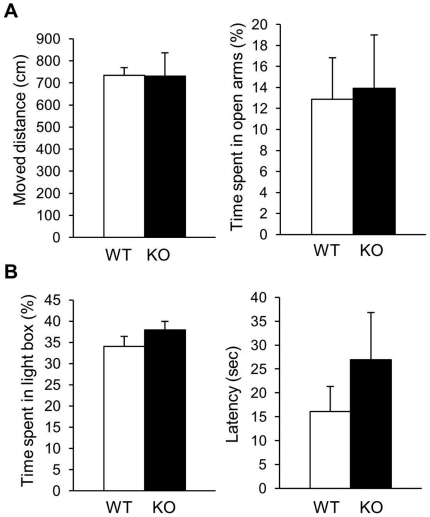
Normal anxiety level of *Slitrk6*-KO mice.

Because *Slitrk6*-KO mice showed altered spontaneous activities in the three different contexts (home cage, OF box, and HB box), we evaluated whether these behavioral abnormalities were caused by elevated anxiety in a novel environment. We therefore performed the elevated plus maze test and light–dark box test, which are commonly used in neurobiological anxiety research. In these tests, however, we did not find any obvious differences between WT and *Slitrk6*-KO mice (Fig. 5). These results indicated that alterations of spontaneous activity in the home cage, OF, and HB tests are due to cognitive dysfunction in the *Slitrk6*-KO mice, rather than elevated anxiety.

The Morris water maze test, fear-conditioning test, forced swimming test, social interaction tests, tail flick test, and hot plate test did not show any abnormalities in the *Slitrk6*-KO mice as compared to the WT (Table 1).

## Discussion

### The *Slitrk6*-KO mouse is a novel animal model of sensorineural deafness

ABRs and ASRs indicated that there is an impaired auditory function in *Slitrk6*-KO mice. A mild (ca. 20-dB) elevation of the sound intensity threshold was observed for 8- and 16-kHz sound stimuli, resulting in a strong decrement of the ABR first wave (peak I). The results suggest that the reduction of peak I to the 80-dB stimulus is related to both the *Slitrk6* loss-of-function and the impaired auditory function. Although the precise origins of ABR waves are not yet well defined, it is generally agreed that the first wave represents activities of the auditory nerve and the late waves represent neural transmission within the central auditory circuit [16]. Our previous study revealed that during embryonic development of *Slitrk6*-KO mice the number of axon projection from the spiral ganglion to cochlear hair and the number of the spiral ganglion neurons were lower than seen in WT mice [5]. The total cell number in the spiral ganglion of *Slitrk6*-KO mice was one half that in WT, and the decreased number of the projection was clearly observed as late as 4 weeks after birth. Therefore, it is likely that the reduction of peak I may reflect these morphological differences in the inner ear neural circuit of *Slitrk6*-KO mice.

It is interesting that elevation of the sound intensity threshold was not observed for the 24-kHz sound stimulus. The frequency-selective sensitivity loss might not be fully explained by the innervation defects in the inner ear, considering that the projection impairment was generally observed from the basal to apical region of the cochlea. It is possible that some mechanism compensates for the desensitization to 24-kHz sounds. The enigmatic peak III increment might reflect such a process.

Many genes are associated with hearing impairment in mice [17], [18], and 44 of them have been linked to human hereditary hearing loss [17]. Our findings indicate that *Slitrk6*-KO mice could be a novel model of sensorineural deafness. According to the current hereditary hearing loss database (http://hereditaryhearingloss.org), the nearest neighboring gene related to hearing impairment is an autosomal dominant nonsyndromic gene (DFNA 33, 13q34-qter) [19] approximately 27 Mb apart from *SLITRK6* (13q31.2). Thus, the involvement of functional deficits in *SLITRK6* in human hereditary hearing impairment awaits further investigation. Furthermore, *Slitrk6* inner ear abnormalities are known to involve altered neurotrophin-signaling [5]. NT-3 delivery has been attempted in model animals [20]–[22] with the aim of clinical treatment of hearing impairment, and it would be beneficial to further clarify the mechanism of *Slitrk6* action in development of the inner ear and auditory signal processing.

### Vestibular function abnormalities in *Slitrk6*-KO mice

*Slitrk6-*KO mice showed moderate deficit in the gain of vVOR, but not in hVOR. Based on the orientations of mouse semicircular canals [23], horizontal semicircular canals are selectively stimulated in the test of hVOR, whereas the anterior and posterior semicircular canals are equally stimulated in the test of vVOR in our assay system. The involvement of otolith in the head rotation used in our vVOR test was small, as the effect of gravity was minimal in the head rotation in nose-up position perpendicular to the earth axis. On the other hand, we previously showed that the neural projection to posterior crista, which develops to posterior semicircular canal, was often lost in *Slitrk6-*KO mice (78.3% at E13.5 [*n* = 83], 85.7% at E15.5 [*n* = 28], and 90.3% after P14 [*n* = 62]), but those to the anterior and horizontal canal cristae and otolith organs remained intact [5]. Thus, the defect of VOR (normal hVOR and impaired vVOR) is consistent with the anomaly of vestibular nerve projection (intact innervation of anterior and horizontal cristae and decreased innervation of posterior crista) in *Slitrk6-*KO mice. Concurrently, the VOR results suggest that function of *Slitrk6-*KO horizontal canal is not obviously impaired, indistinguishable from those of WT. However, the functional and structural properties of the remained inner ear neural circuits of *Slitrk6-*KO should be more carefully examined because embryonic *Slitrk6* expression broadly occurs in the inner ear sensory epithelia [5].

Many mutant mice with vestibular function abnormalities are listed in the current mouse genome informatics database (http://www.informatics.jax.org/). However, we did not find any mice with vertical-direction limited VOR abnormalities. In this regard, the vestibular dysfunction in *Slitrk6*-KO mice is unique. Together with the selective innervation defects to the posterior crista, *Slitrk6*-KO mice could serve as a useful model to study the role of the semicircular canal in vestibular function.

Although the vestibular function alteration was clear in the vVOR, it is unclear how the other behavioral phenotypes in *Slitrk6*-KO mice reflect the altered vestibular function. For example, *Slitrk6*-KO mice showed a significant decrement in the immobility time of the tail suspension test (Table 1). This alteration could indicate the absence of a normal vestibular response for vertical head movement. However, even if this is the case, the overall vestibular function deficit appears to be mild and selective. The vestibular innervation defects in the posterior canal may be partially compensated by the anterior semicircular canal and possibly the otolith organs, which also sense vertical head movements.

In humans, a posterior semicircular canal dysfunction is related to benign paroxysmal positional vertigo, in which displaced otoconia are thought to enter a semicircular canal, usually in the posterior one [24], [25]. Although this is a disorder of late onset (mean onset 54 years old) [24], there are pediatric patients with balance and vestibular disorders [26], [27]. Considering that significant associations exist between sensorineural hearing loss and balance disorders [26], it is possible that *SLITRK6* is involved in some pathophysiological processes of pediatric vestibular disorders.

### Cognitive dysfunction of *Slitrk6*-KO mice

This study revealed novel behavioral phenotypes of *Slitrk6*-KO mice (Table 1). Among them, the abnormalities revealed by the OF and HB tests may reflect similar aspects of altered higher brain function in *Slitrk6*-KO mice. In both tests, the abnormalities appeared soon (within 5 min) after entry into the test boxes. Therefore, we assume that the novel environments induced the differential behaviors. Our findings clearly indicated a neurological basis for these behavioral abnormalities, which appeared to reflect increased attention to the environment and emotional responses similar to “restlessness” or “confusion.”

It is tempting to correlate these behavioral abnormalities with the role of *Slirk6* in the thalamus, where its expression is clearly demarcated among regions of the central nervous system [1], [4], [6]. If we assume that the thalamic expression is significant, some sensory information processing during adaptive behaviors may be dysfunctional in *Slitrk6*-KO mice, considering the many roles played by the thalamus in relaying and modulating sensory signals [28]–[30]. Future studies should examine whether *Slitrk6* loss-of-function impairs the development or the function of the thalamic neural circuit. Clarification of the molecular function of *Slitrk6* will contribute to a better understanding of the higher function of the mammalian brain.

## Materials and Methods

### Animals

Animal experiments were approved by the Animal Experiment Committee of the RIKEN Brain Science Institute (approval no. H18-2B032), and the mice were maintained at the institute's Research Resource Center. The mice were kept on a 12-h light–dark cycle, with the dark cycle occurring from 20:00 to 08:00. All the testing described here was performed with adult male mice. The behavioral tests were started when the mice were 12 weeks old and completed before they reached the age of 16 weeks. In total, 45 pairs of WT and KO mice were used in this study.

### Auditory brainstem response

For the measurement of ABRs, mice were anesthetized with an intraperitoneal injection of 60 mg/kg sodium pentobarbital (Nembutal, Dainippon Pharmaceutical Co., Ltd., Osaka, Japan), and needle electrodes were inserted at the vertex and pinna with a ground near the tail. ABRs were evoked with 4-ms tone pips at 40 per second with a 0.4-ms cosine squared rise–fall envelope and alternating in polarity to remove frequency-following responses. The voltage difference between the pinna and vertex was amplified (10,000×), filtered, digitized at 100 kHz, and averaged across 512 presentations. The sound level was decreased in 10-dB steps from an 80-dB sound pressure level. The threshold, amplitude, and latency of responses were defined by visual inspection of stacked waveforms.

### Auditory startle response

In this test, each mouse was put into a small cage (30 or 35 mm diameter, 12 cm long) that was set on a sensor block within a sound-proof chamber (60×50×67 cm [height]). A dim light was equipped on the ceiling of the sound-proof chamber (10 lux at the center of the sensor block), and a 65-dB white noise was presented as background noise. In the ASR test, each mouse was acclimatized to the experimental condition for 5 min, then the experimental session began. In the first session, a 120-dB startle stimulus (40 ms) was presented to the mouse 10 times at random intertrial intervals (10–20 s). In the second session, the startle response to stimuli at various intensities was assessed. Five times of 70 to 120 dB (70, 75, 80, 85, 90, 95, 100, 110, 120 dB) white noise stimuli (40 ms) were presented in quasi-random order and random inter-trial intervals (10–20 sec). In the prepulse inhibition session, the mouse experienced five types of trials: (1) no stimulus; (2) startle stimulus (120 dB, 40 ms) only; (3) 70-dB prepulse (20 ms, lead time 100 ms) and 120-dB pulse; (4) 75-dB prepulse (20 ms, lead time 100 ms) and120-dB pulse; and (5) 80-dB prepulse (20 ms, lead time 100 ms) and 120-dB pulse. Each trial was repeated 10 times in quasi-random order at random intertrial intervals (10–20 s). In the final session, again a 120-dB startle stimulus (40 ms) was presented to the mouse 10 times at random intertrial intervals (10–20 s). The total duration of an ASR test was about 35 to 40 min. After each trial, holding chambers were washed with tap water, wiped with a paper towel, and dried. Apparatuses and software for data analysis used were commercially available ones (Mouse Startle, O'Hara, Tokyo, Japan).

### Vestibulo-ocular reflex and optokinetic response eye movements

Eye movement was measured by the infrared TV method as described previously [31], [32]. Under isofluorane (Escain, Mylan-Japan, Tokyo, Japan) anesthesia and aseptic conditions, a platform for head fixation was made on the mouse cranial bone using synthetic resin (Superbond C &B, Sun Medical, Tokyo, Japan) and one 15-mm stainless bolt. Two days after surgery, a mouse was mounted on the turntable surrounded by a checked-pattern (check size, 4°) screen (diameter, 60 cm; height, 60 cm), with the head fixed and the body loosely restrained in a plastic cylinder. The horizontal and vertical vestibulo-ocular reflexes (hVOR and vVOR, respectively) and horizontal optokinetic response (hOKR) eye movements were measured. The hVOR was tested in the dark by sinusoidal turntable oscillation at a frequency of 0.11–0.50 Hz and peak-to-peak amplitude of 10° on the plane parallel to the bilateral horizontal semicircular canals. The hOKR was tested in the light with sinusoidal screen oscillation at a frequency of 0.11–0.33 Hz and peak-to-peak amplitude of 10–20° (maximum screen velocity, 3.5–10.5°/s) on the same plane. The vVOR was tested in the dark with turntable oscillation at a frequency of 0.11–0.5 Hz and peak-to-peak amplitude of 10° on the plane perpendicular to the plane of the bilateral horizontal semicircular canals. During the test of vVOR, the mouse laid on its back in the plastic cylinder with the head-up position on the turntable [33]. More than 10 cycles of the evoked horizontal (hVOR and hOKR) or vertical (vVOR) eye movements free from artifacts due to blinks and saccades were averaged, and the mean amplitude and phase were calculated by a modified Fourier analysis [34]. A gain of the eye movement was defined as the ratio of the peak-to-peak amplitude of eye movements to that of the turntable or screen oscillation. The phase was defined as 0° when the peak of the eye movement was opposite to the peak of turntable oscillation in the hVOR and vVOR and when the peak of the eye movement matched the screen oscillation in the hOKR.

### Rota-rod test

A mouse was placed on a rotating rod (O'Hara) and the time it was able to maintain its balance walking on top of the rod was measured. The speed of rotation was 4 rpm on day 1, and the speed was accelerated from 4 to 40 rpm over a 4-min period and then maintained at 40 rpm for another 1 min on days 2 to 5. Mice were tested in one trial for 2 min on day 1 and in four trials with a maximum time of 300 s (intertrial intervals were 20–30 s) on days 2 to 5. The time between placement and falling off or revolving around the rod was recorded manually.

### Home cage activity

Spontaneous activity of each mouse in its home cage was measured using a 24-channel ABsystem 4.0 (Neuroscience, Tokyo, Japan). Cages were individually set into the stainless steel compartments of a negative breeding rack (JCL, Tokyo, Japan). An infrared sensor was equipped on the ceiling of each compartment and it detected movements of the mice. Home cage activity was measured for 1 week, starting from the afternoon of the day mice were transferred to the behavioral laboratory (day 1). Only data from days 2 to 8 were statistically analyzed. After termination of the home cage activity measurements, cages and bedding materials were changed and the mice were maintained in a micro-isolation rack (Allentown, PA, USA) throughout the behavioral screening.

### Hole-board test

For this test, an OF system made of gray plastic (50×50×40 cm [height]) with four equally separated holes (3 cm diameter, each with an infrared sensor) on the floor was used (model ST-1/WII, Muromachi-kikai, Tokyo, Japan). The field was illuminated by fluorescent light (180 lux at the center of the field), and the level of background noise was approximately 50 dB. The behavior of each mouse was monitored by a CCD camera (placed about 1.5 m above the field). In the HB test, a mouse was introduced into the center of the field and allowed to explore freely for 5 min. Total moving time (s), distance traveled (cm), latency for head dipping (s), number of head dips, duration of head dipping (s), duration of rearing (s), and number of rearing episodes were measured as indices. Data were collected and analyzed using CompACT VAS system (Muromachi-kikai).

### Open field, elevated plus maze, and light–dark box tests

Open field, elevated plus maze, and light–dark box tests were performed as described previously [35].

### Statistical analyses

Statistical analyses were conducted using the SPSS statistical package (ver. 16.0, SPSS Japan Inc., Tokyo, Japan). Parametric data were analyzed by Student's *t*-test, and nonparametric data were analyzed by Mann-Whitney's *U*-test. Reported *P* values refer to Student's *t*-test unless otherwise noted. Effects of factors were analyzed by one-way ANOVA, two-way ANOVA with post hoc tests, and generalized linear model. Differences were defined as statistically significant when *P*<0.05.
